# Mask-Aware Spatiotemporal Classification of Millimeter-Wave Radar Point Cloud Sequences Using DGCNN and Transformer for Child–Pet Recognition in Enclosed Spaces

**DOI:** 10.3390/s26051580

**Published:** 2026-03-03

**Authors:** Yehui Shi, Jianhong Shi

**Affiliations:** State Key Laboratory of Advanced Optical Communication Systems and Networks, Institute of Quantum Sensing and Information Processing, School of Sensing Science and Engineering, Shanghai Jiao Tong University, Shanghai 201100, China; s82191943@sjtu.edu.cn

**Keywords:** FMCW, sparse point cloud, target classification, fusion of spatiotemporal features, attention mechanism

## Abstract

Applications in enclosed spaces such as vehicle cabin on-site detection, human–pet separation, and pet care have put forward higher requirements for non-contact target recognition. Millimeter-wave radar point clouds have advantages such as privacy friendliness and robustness against low light and occlusion. However, their point clouds are generally sparse, with obvious noise and multipath interference. Moreover, the fluctuation of point numbers over time makes alignment and feature learning difficult, which leads to performance degradation of existing point cloud classification methods in complex environments. To this end, this paper proposes a spatiotemporal joint classification framework for millimeter-wave point cloud sequences: An effective point mask mechanism is introduced in the spatial dimension to suppress the interference of invalid points generated by alignment on the neighborhood composition and feature aggregation and improve the reliability of local geometric representation; and to integrate attention-based time series modeling in the time dimension and enhance category separability by using cross-frame dynamic patterns. The experimental results show that the proposed method can achieve an accuracy rate of 97.8% in the three-classification tasks of Child, Cat and Dog and the ablation analysis verifies the key contributions of the mask mechanism and time series modeling to robust recognition. This framework provides a deployable and more generalized millimeter-wave point cloud solution for the identification of life forms in confined spaces.

## 1. Introduction

With the development of intelligent cockpits and indoor intelligent monitoring, the demand for presence perception and differentiation of life forms in enclosed spaces is constantly increasing. Typical applications include child presence detection (CPD) in vehicle cabins, occupant classification and safety alerts in vehicles, etc. [1,2,3]. At the engineering implementation level, the industry and research community generally focuses on the solution approach of “concealed installation, all-weather operation, and minimal reliance on cameras”. Millimeter-wave radar, due to its privacy-friendly nature, insensitivity to light, and certain penetration/anti-occlusion capabilities, has gradually become an important candidate sensor for in-vehicle perception [4,5,6].

Regarding in-vehicle occupancy detection and occupant classification, existing studies have proposed various in-cabin detection frameworks and algorithmic routes based on millimeter-wave radar, including in-cabin occupancy detection for complex driving scenarios and seat-level occupancy/classification determination, providing an application basis and feasibility for CPD and human–pet separation [7,8]. However, from the perspective of method mechanisms, a considerable proportion of existing studies still rely more on vital sign information (such as breathing and heartbeats) for detection or recognition. Such methods can often obtain relatively clear periodic components when the target is relatively static, but in real vehicle cabins or indoor environments, children and pets do not remain static all the time. Body movement components can cover or significantly distort the weak periodic components of vital signs, making feature separation and stable extraction difficult, and leading to insufficient robustness of static vital sign-driven strategies under dynamic conditions. A large number of studies have systematically discussed the suppression of body movement interference, motion compensation, and robust vital sign extraction from the perspectives of algorithms and signal processing, which also indirectly indicates the objective limitation that “relying solely on vital signs is not robust in dynamic scenarios” [9,10,11,12,13]. At the same time, millimeter-wave radar point clouds are sparser than laser point clouds, with the number of points fluctuating significantly per frame and being more susceptible to noise, multipath interference, and clutter, resulting in insufficient stability and separability of single-frame geometric shapes. Recent public datasets and benchmark studies have also generally emphasized the data attributes of “sparsity and stronger noise” of millimeter-wave point clouds and their challenges to learning algorithms [14,15,16]. Additionally, deep learning methods typically require fixed-shape inputs, and point cloud alignment (truncation/filling) is inevitable. Once invalid points participate in k-nearest neighbor graph construction and feature aggregation, they can disrupt the true local topology and introduce incorrect statistics, further weakening the spatial representation ability. Considering that the morphological differences between categories such as cats and dogs are limited in a single frame, differentiation often relies on cross-frame dynamic patterns and motion rhythms, thus requiring modeling methods that can handle dynamic targets, integrate spatiotemporal information, and be more robust to sparsity and noise. The development of point cloud deep learning and attention-based sequence modeling provides technical foundations and theoretical support for this [17,18,19,20].

This paper proposes a spatiotemporal joint classification network for millimeter-wave point cloud sequences to achieve reliable classification and differentiation of dynamic targets. The network adopts local neighborhood-based topological feature learning in the spatial dimension to describe the geometric relationship of point clouds and introduces an effective point masking mechanism to suppress the interference of invalid points on neighborhood graph construction and feature aggregation during alignment, thereby alleviating the structural distortion caused by the fluctuation of point cloud points in sparse point clouds. In the temporal dimension, it combines attention-based temporal modeling to integrate cross-frame dynamic changes and enhance the separability of similar categories. Through comparative and ablation experiments, this paper verifies the key contributions of effective point suppression and temporal modeling to performance improvement, providing more robust and deployable algorithmic support for applications such as CPD and human–pet separation in enclosed spaces.

## 2. The Principle of FMCW Millimeter-Wave Radar

### 2.1. The Measurement Principle of Millimeter-Wave Radar

Frequency-Modulated Continuous-Wave (FMCW) radar is widely used in automotive and consumer electronics due to its ability to measure distance, speed and angle. The isolation degree between the transmitting (Tx) and receiving (Rx) subsystems of an FMCW radar system directly affects the system’s performance, especially the target detection sensitivity. The FMCW radar front end includes a transmitter and a receiver. The transmitted signal (*S_TX_*) is described as follows:

(1) $$s_{TX}\left(t\right)=A_{TX}\cos\left[\int \left(\omega_{0}+A_{b}*t\right)dt\right]\;=A_{TX}\cos\left[\left(\omega_{0}+\frac{A_{b}}{2}*t\right)*t\right]$$

After a delay of $T_{d}$, the reflected signal is received, which is expressed as:

(2) $$s_{RX}\left(t\right)=\beta *s_{TX}\left(t-T_{d}\right)\;=A_{RX}\cos\left[\left(\omega_{0}+\frac{A_{b}}{2}*\left(t-T_{d}\right)\right)*\left(t-T_{d}\right)\right]$$

**Figure 1:**
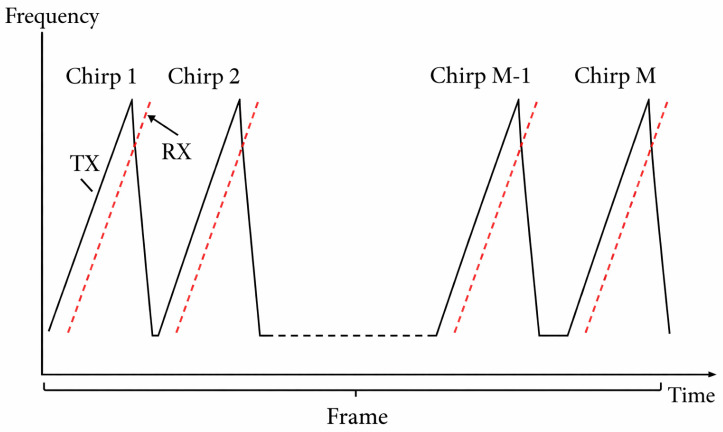
Tx/Rx signals.

β represents the loss of the signal during the transmission process. Figure 1 shows the transmitted signal and the received signal.

The intermediate frequency (IF) signal is generated by mixing the transmitted signal with the received signal, during which the carrier is eliminated. This process is known as “demodulation” or “decompression”. The expression of the “sum frequency signal” generated at the output end of the mixer is:

(3) $$s_{IF}\left(t\right)=s_{RX}\left(t\right)*s_{TX}\left(t\right)\;=\frac{A_{TX}*A_{RX}}{2}\left[\cos\left(2*\omega_{0}*t+A_{b}*t^{2}-A_{b}*T_{d}*t+\frac{A_{b}}{2}*T_{d}^{2}-\omega_{0}*T_{d}\right)+\cos\left(A_{b}*T_{d}*t+\omega_{0}*T_{d}-\frac{A_{b}}{2}*T_{d}^{2}\right)\right]$$

The first term represents the high-frequency component, which has been filtered out, leaving the beat frequency signal related to the target distance. The second cosine function describes the beat frequency signal at a fixed frequency. In (4), $T_{d}=2*\frac{R}{c}$, where R is the target distance, and $S=\frac{A_{b}}{2\pi }$ defines the frequency modulation slope. According to this formula, the beat frequency of the intermediate frequency signal is related to $T_{d}$.

(4) $$f_{IF}=S*T_{d}$$

The distance to the target is determined by analyzing the frequency of the IF signal, and this process is achieved through a one-dimensional Fast Fourier Transform (1D-FFT). The output of the 1D-FFT provides the spectral features necessary for estimating distance, velocity and angle. Velocity information is derived by applying a second Fourier transform to the 1D-FFT data, and the beamforming algorithm also utilizes these data. For multiple reflections, the IF signal is:

(5) $$s_{IF}\left(t\right)=\underset{k=0}{\sum }A_{k}*\cos\left(2*\pi *f_{k}*t+\emptyset_{k}+noise\right)$$

Here, k represents the k different reflected signals received by the receiver. $A_{k}$ denotes the reflected energy of each target, while $\emptyset_{k}$ is the phase difference between the transmitted signal and the received signal.

This radar system is also capable of monitoring phase changes in the intermediate frequency signa. The phase ϕ of the received signal can be expressed as:

(6) $$\phi \left(t\right)=\frac{4\pi d\left(t\right)}{\lambda }$$

Here, λ represents the wavelength of the radar signal and d(t) indicates the displacement that varies with time. The phase information is extracted by applying a 1D-FFT to the intermediate frequency signal in the time domain, where the in-phase (I) and quadrature (Q) components are used for phase demodulation:

(7) $$\phi \left(t\right)=\arctan\left(\frac{Q}{I}\right)$$

However, if the power is transmitted from the transmitter to the receiver due to the relatively small propagation delay, a nearly DC spectrum will be generated in the intermediate frequency domain, which is known as the DC offset problem. This issue affects the DC level and noise floor of the entire spectrum, meaning that the intermediate frequency signal only contains the coupling energy within the chip. Due to the spectral leakage produced by the Fast Fourier Transform (FFT), the higher the DC level, the greater the values at other frequencies. Therefore, the coupled power increases the DC and other frequency spectra, thereby raising the noise floor of the entire system. The energy transmitted from the transmitter to the receiver mainly increases the low-frequency noise, thereby raising the noise level throughout the one-dimensional FFT spectrum. Theoretical analysis indicates that the increase in the DC level directly affects the performance of the FMCW radar system.

### 2.2. The Formation Mechanism of Point Cloud Signals

**Figure 2:**
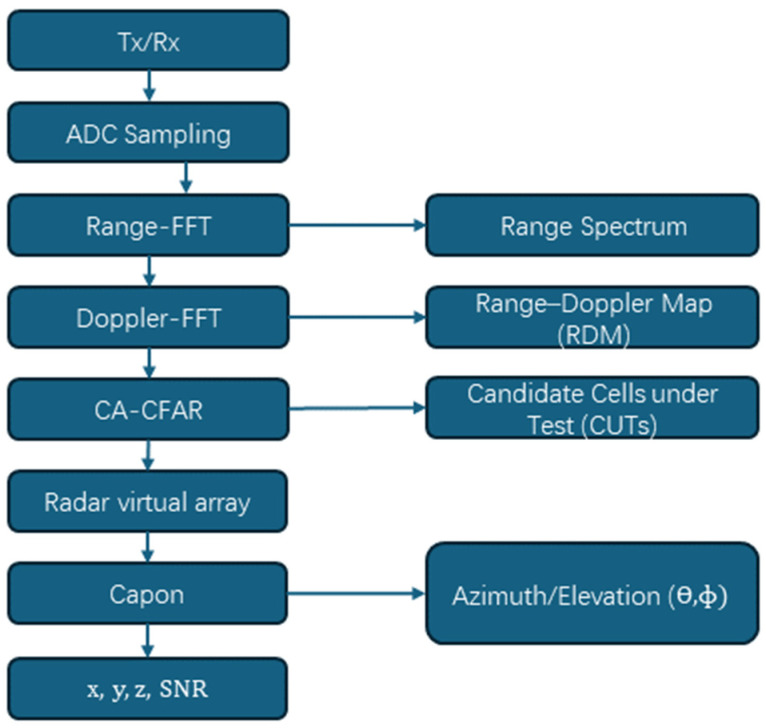
Radar point cloud generation flowchart.

This paper adopts the TI IWR6843AOP millimeter-wave FMCW radar (Texas Instruments, Dallas, TX, USA) for data acquisition. The system uses three transmitting antennas and four receiving antennas (3Tx × 4Rx), and switches the transmitting channels in time division within one frame through the time-division multiplexing multiple-input and multiple-output (TDM-MIMO) method to form a virtual array observation. The point cloud is output at a frame period of $T_{f}\;=\;200\;\mathrm{ms}$, corresponding to a point cloud frame rate of 5 Hz. Each frame is configured with NumLoops = 32, indicating that each Tx transmits 32 chirps within one frame. Figure 2, shows the process of generating point clouds by the millimeter-wave radar. After the FMCW linear frequency modulated chirp is transmitted, the target echo is mixed with the local oscillator signal to obtain the beat signal, which is then sampled by the analog-to-digital converter (ADC) after low-pass filtering. For a target at a distance of R, its beat frequency approximately satisfies:

(8) $$f_{b}\approx ST_{d}=S\frac{2R}{c},\;R=\frac{c}{2S}f_{b}$$

Here, S represents the chirp slope, $T_{d}$ is the round-trip time delay, and **c** is the speed of light. This relationship indicates that the target distance information is mapped onto the beat frequency signal frequency. A one-dimensional FFT is performed on the fast-time sampling sequence of each chirp to obtain the range spectrum and form discrete range bins. In this paper, the chirp sweep bandwidth is B = 2 GHz, and the theoretical range resolution is:

(9) $$\Delta R=\frac{c}{2B}=7.5\;\mathrm{cm}$$

To focus on the effective detection area and reduce the subsequent computational load, only 64 distance bins are retained as the distance ROI in the offline data processing/feature construction. The slow-time sequence across chirps is subjected to two-dimensional FFT to obtain the range–Doppler map, where each cell corresponds to the energy response of a certain range and a certain radial velocity, providing input for subsequent target detection. To maintain a stable false alarm rate under varying noise and clutter levels, Cell-Averaging Constant False-Alarm Rate (CA-CFAR) [20] is adopted on the range–Doppler map for adaptive threshold detection. For the cell under test (CUT) [21], the noise power *P_n_* is estimated using the neighboring training cells, and the detection threshold T is calculated as follows:

(10) T=αPn

Here, α is determined by the set false alarm probability P_fa_ and the number of training units. In this paper, P_fa_ is set to 0.005. When the CUT energy exceeds the threshold T, it is judged as a candidate target unit, and the index of the candidate point and the related intensity information are output. Since the point cloud only outputs the detection points that pass through CFAR, the point cloud signal-to-noise ratio (SNR) is often positive or non-negative, which is the result of the detection threshold screening. To reduce multiple counts caused by side lobes or repeated peaks in the neighborhood, peak grouping or non-maximum suppression (NMS) is performed on the CFAR candidate points to retain the local main peak, making the output target points more stable and the number more reasonable. For each candidate target unit, the corresponding complex observations are extracted on the 3Tx × 4Rx virtual array channels to form an array observation vector:

(11) $$x\in C^{M},\;M=3\times 4=12$$

This paper employs Capon (Minimum Variance Distortionless Response, MVDR) spectral estimation for the estimation of the angle of arrival (AoA). The covariance matrix is estimated based on the observation vector (or local snapshot set):

(12) $$R=E\left[xx^{H}\right]$$

The Capon spatial spectrum is:

(13) $$P_{Capon}\left(\theta ,\phi \right)=\frac{1}{a^{H}\left(\theta ,\phi \right)R^{-1}a\left(\theta ,\phi \right)}$$

Here, $a\left(\theta ,\phi \right)$ is the array steering vector. The target azimuth/elevation estimation is obtained by searching for the spectral peak on the angle grid. Compared with the traditional FFT beamforming, Capon has a stronger ability to suppress side lobes and is more suitable for indoor multipath and clutter environments. Combining the distance estimation R and the angle estimation $\left(\hat{\theta },\hat{\phi }\right)$, the spherical coordinates are converted to rectangular coordinates to obtain the three-dimensional coordinates:

(14) $$x=R\cos\hat{\phi }\cos\hat{\theta },\;y=R\cos\hat{\phi }\sin\hat{\theta },\;z\;=\;R\sin\hat{\phi }$$

Meanwhile, the SNR is calculated based on the intensity of the detection unit and the noise estimation (in engineering implementation, it can be converted from the peak value of the CFAR output and the noise estimation). Finally, the point cloud set of each frame is output:

(15) $${\left\{\left(x_{i},y_{i},z_{i},{\mathrm{SNR}}_{i}\right)\right\}}_{i=1}^{N}$$

And it is continuously output at a period of $T_{f}=200\;\mathrm{ms}$.

## 3. DGCNN-Transformer Network Architecture

### 3.1. Neural Network Structure Diagram and Overall Framework Description

**Figure 3:**
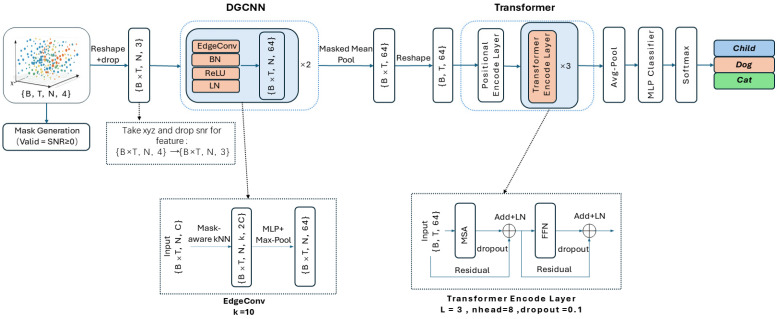
Neural network architecture. MSA denotes multi-head self-attention, FFN denotes feed-forward network, and Add + LN denotes residual addition followed by LayerNorm.

As shown in Figure 3, the radar point cloud sequence classification network proposed in this paper is composed of a spatial feature extraction module (DGCNN) and a temporal modeling module (Transformer Encoder) in series. The model input is a continuous sequence of T = 15 frames of millimeter-wave point clouds, with each frame uniformly sampled/filled to N = 150 points, and each point containing four-dimensional attributes (x, y, z, SNR). The network first extracts the local geometric features of the point cloud at the single-frame scale using DGCNN, then obtains the global representation of each frame through frame-level aggregation, and subsequently models the cross-frame temporal dependencies using multiple layers of the Transformer Encoder. Finally, it outputs a three-classification result (Child-sized mannequin, Dogs, Cats) through a multi-layer perceptron (MLP).

#### 3.1.1. Input Representation and Valid Point Mask

Let the input tensor of a batch be $X\in R^{B\times T\times N\times 4}$, where the four channels correspond to $\left(x,y,z,\mathrm{SNR}\right)$. Due to the fact that the number of points in each frame of the actual point cloud is not fixed, this paper aligns each frame of the point cloud to a fixed length. Millimeter-wave point clouds have the characteristics of sparsity, large fluctuations in the number of points, and many noise points. According to sample statistics, the median number of points per frame of the point cloud is approximately 152 points/frame. Considering the computational efficiency and the need for batch alignment, this paper takes N = 150 as the fixed number of points: when the number of points is greater than N, down-sampling is performed; when the number is less than N, padding is used to complete (the SNR of the padding points is −1). If the graph is directly constructed on all points, the padding points may be wrongly selected as neighbors, thus destroying the local geometric structure. Therefore, this paper introduces an effective point mask in the kNN neighborhood search to exclude invalid points from the neighbor candidate set; in the subsequent feature aggregation, only the effective points are counted, thereby avoiding the interference of pseudo points. This paper constructs the effective point mask $m_{t,i}=I\left(SNR\geq 0\right)$, where $m_{t,i}=1$ indicates an effective point and $m_{t,i}=0$ indicates a padding point. It should be noted that in the spatial feature learning stage, only the (x, y, z) three-dimensional coordinates are used as the geometric input, and SNR is only used to generate the mask and remove invalid points in the kNN graph construction and feature aggregation. To facilitate frame-by-frame spatial encoding, the input sequence is first reshaped to $R^{\left(B\cdot T\right)\times N\times 4}$, and then the three-dimensional coordinates are taken to form $R^{\left(B\cdot T\right)\times N\times 3}$ as the input of DGCNN. This mask strategy can still stably construct the graph in frames with insufficient points or few effective points, while removing pseudo points, ensuring that the local graph structure more truly reflects the geometric neighborhood relationship.

#### 3.1.2. Spatial Feature Extraction: Mask-Aware DGCNN

This paper adopts the EdgeConv structure of DGCNN to model the local geometric topology of single-frame point clouds. For each point i, a neighborhood set $N\left(i\right)$ is first constructed based on mask-aware kNN (k = 10), and invalid points $(m_{t,i}\;=\;0)$ are excluded from the candidate set during neighbor selection to prevent padding points from entering the neighborhood graph. It should be noted that the first layer of EdgeConv constructs the graph in the coordinate space (x,y,z), and the second layer dynamically reconstructs the neighborhood in the feature space of the output from the previous layer, thereby capturing higher-order local structure patterns layer by layer. EdgeConv first constructs edge features:

(16) $$e_{ij}=h_{\Theta }\left(\left[x_{i},x_{j}-x_{i}\right]\right),\;\;j\in N\left(i\right)$$

Here, $x_{I}$ represents the feature of the central point, and $x_{j}-x_{I}$ is the relative displacement. $H_{\Theta }$ denotes the shared MLP, which is implemented by a 1×1 convolution, BatchNorm, and ReLU. This edge feature design retains both the information of the central point and the relative geometric relationship: $x_{j}-x_{I}$ emphasizes the relative geometric structure within the neighborhood, showing stronger translation invariance; while $x_{I}$ provides local context, enabling the network to distinguish structural differences such as dense and edge regions in the point cloud, and is suitable for characterizing discriminative features such as “target posture + density/structural changes” in millimeter-wave point clouds. Subsequently, the edge features within the neighborhood are symmetrically aggregated (Max Pooling) to obtain the updated point features:

(17) $$x_{i}^{\prime }=\underset{j\in N\left(i\right)}{\max}e_{ij}$$

This paper stacks two layers of EdgeConv and introduces LayerNorm after each layer to enhance the training stability. After two layers of spatial encoding, the expanded frame-by-frame and point-by-point feature representation is:

(18) $$F\in R^{\left(B\cdot T\right)\times N\times D},\;D=64$$

#### 3.1.3. Frame-Level Aggregation

To map the point-wise features to frame-level global representations, this paper adopts masked mean pooling to average the valid point features of each frame:

(19) $$g_{t}=\frac{1}{\sum_{i=1}^{N}m_{t,i}}\sum_{i=1}^{N}m_{t,i}f_{t,i}$$

Here, $f_{t,i}$ represents the feature of the i-th point in the t-th frame (t indexes the frame and i indexes the point within the frame). This strategy ensures that invalid points do not affect the frame representation and mitigates the scale bias caused by the fluctuation in the number of valid points across different frames. After aggregation, a frame-level vector $G\in R^{\left(B\cdot T\right)\times D}$ is obtained, which is then reshaped into a sequence form $R^{B\times T\times D}$ to serve as the input for the temporal modeling module.

#### 3.1.4. Temporal Modeling with Transformer Encoder

To depict the motion patterns and inter-frame dependencies of the target across consecutive frames, this paper adds positional encoding to the frame-level vector sequence $G\in R^{B\times T\times D}$ and inputs it into the Transformer Encoder for temporal modeling. This paper stacks L = 3 Transformer Encoder Layers, each containing multi-head self-attention (nhead = 8) and an FFN, and adopts residual connections and LayerNorm to stabilize training; dropout is set to 0.1. The output of the Transformer is $Z\in R^{B\times T\times D}$. After the Transformer output, this paper performs average pooling along the time dimension to obtain the sequence-level global representation:

(20) $$z=\frac{1}{T}\overset{T}{\underset{t=1}{\sum }}Z_{:,t,:}\in R^{B\times D}$$

Here, $Z_{:,t,:}$ denotes selecting all indices along that dimension.

Then, an MLP classifier (including nonlinearity and dropout) is input, and the three-class logits $y\in R^{B\times 3}$ are output. During the training phase, the classification loss is directly calculated using the logits; during the inference phase, softmax is applied to the logits to obtain the category probabilities, and the final output is the prediction results of the three categories: Child-sized mannequin, Dogs, and Cats. The loss function, optimizer, number of training epochs, data augmentation, and other hyperparameter settings used in the network training will be uniformly provided in the experimental section of Section 4.

## 4. Radar Signal Processing and Experimental Details

### 4.1. Radar Parameter Settings

This paper adopts the TI IWR6843AOP millimeter-wave FMCW radar to collect point cloud data. The point cloud generation link follows the processing flow of “Range-FFT → Doppler-FFT → CA-CFAR → Peak grouping → Capon (MVDR) AoA → Coordinate inversion”, and the theoretical basis is presented in Section 2. This section only provides the key configuration parameters used in the experiments of this paper (Table 1) to ensure reproducibility.

### 4.2. Data Collection

#### 4.2.1. Experimental Environment Setup

**Figure 4:**
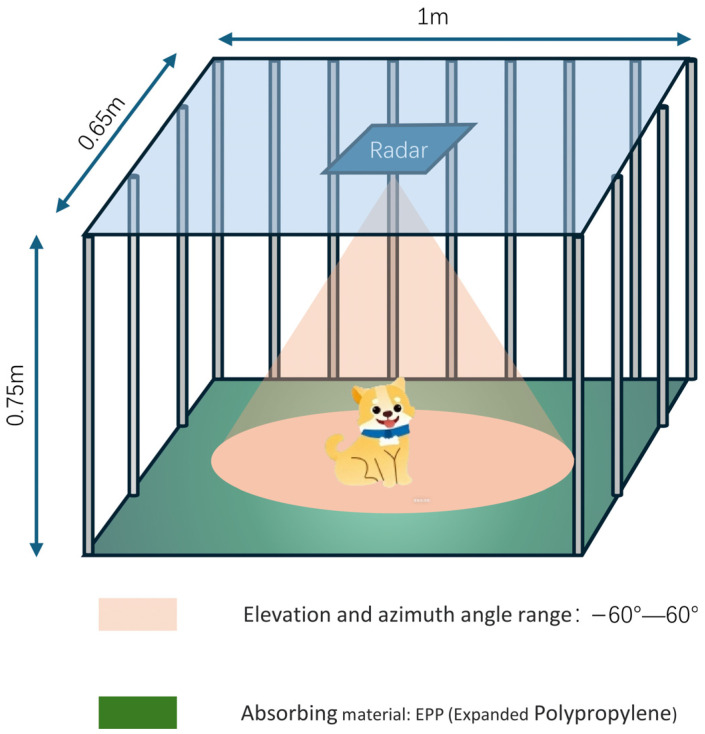
Experimental setting.

Figure 4 shows the experimental setup, which is a metal cage $\left(1.0\;m\;\times \;0.65\;m\;\times \;0.75\;m\right)$. The millimeter-wave radar is installed at the center of the cage roof and works downward, with an azimuth/elevation field of view of approximately −60° to 60°. EPP absorbing material is laid on the cage floor to suppress ground and environmental clutter and improve the stability of point cloud observation. To analyze the main sources of point cloud clutter in an empty cage, this paper first conducts an electromagnetic mechanism analysis of the grid structure of the metal door of the cage. The metal cage door is usually composed of multiple vertical metal columns, whose geometric dimensions are significantly larger than the millimeter-wave wavelength. According to the propagation characteristics of geometric diffraction/mirror reflection, when the millimeter-wave is incident on the surface of the vertical metal column, the energy is mainly scattered along the column direction and propagates downward, making it difficult to effectively return along the original incident path to the receiving antenna. To verify this conclusion, a simplified model of a single cylindrical metal rod (0.5 m in length and 10 mm in diameter) and the transmitting and receiving antennas is established in the electromagnetic simulation software, and the power response at the receiving end is observed. The simulation results show that the echo power at the receiving end is approximately 0, indicating that the metal columns of the cage door are not the main source of noise in the empty-cage environment.

After excluding the significant echo contribution of the metal columns, the stray effective points in the point cloud of the empty cage are more likely to come from strong reflections and multipath effects caused by the cage floor or ground and nearby fixed structures. Based on the above judgment, this paper only lays absorbing material on the cage floor to suppress the energy from the bottom reflection path.

**Figure 5:**
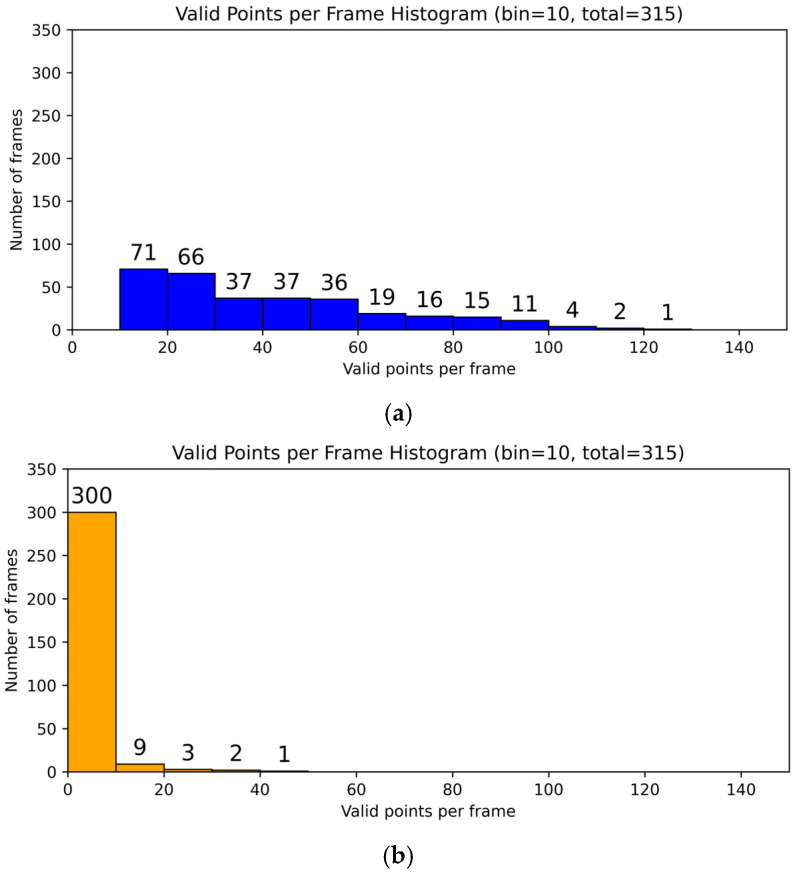
Histograms of the number of valid points per frame in an empty cage: (**a**) without EPP and (**b**) with EPP, where EPP denotes the absorbing material laid on the cage bottom. The horizontal axis indicates the number of valid points detected in each radar frame, and the vertical axis indicates the number of frames falling into each bin. The bin width is 10 points. The value “total” denotes the total number of frames included in the statistics.

Figure 5a,b show the distribution of valid points per frame in the empty-cage condition before and after laying the absorbing material. After applying EPP, the distribution shifts toward the low point-count range and the occurrence of medium-to-high point-count frames is largely suppressed. This trend suggests that the absorbing material mitigates clutter points induced by floor reflection and multipath effects that can pass CFAR detection, thereby reducing background fluctuations and stabilizing the point cloud baseline under empty-cage measurements.

#### 4.2.2. Data Preprocessing

To adapt to the batch training of deep networks and reduce the impact of sparsity and noise in millimeter-wave point clouds on modeling, this paper performs region of interest (ROI) screening, fixed-point number alignment, and windowing grouping on the point cloud data to form a unified input format. Firstly, the point cloud in this paper is output by the signal processing chain. Since CFAR screens out low-energy cells with a constant false alarm threshold, the remaining detection points correspond to the cells “passing the threshold”, and their output SNR is non-negative in the implementation. Therefore, this paper takes SNR ≥ 0 as the criterion for valid points and constructs a valid point mask $m_{t,i}=I\left(\mathrm{SNR}\geq 0\right)$. When the number of points in a frame is insufficient for alignment, padding is used to complete it, and the padded points are uniformly assigned SNR = −1, so that invalid points can be explicitly excluded in subsequent processing through the mask to avoid interference from false points. Secondly, considering the size of the experimental device and the installation position of the radar, to suppress ground/cage bottom multipath and occasional stray points from high places, this paper only retains points that satisfy 0 < z < 0.75 m as valid measurement range data, and directly discards points outside the ROI. Subsequently, to achieve tensorized input, this paper uniformly aligns each frame of point cloud to N = 150 points and the median in statistics is approximately 152 points/frame, taking the approximate value for alignment: when the number of points is greater than N, down-sampling is performed; when it is less than N, padding is used to complete it. In the time dimension, the system frame rate is 5 fps, and T = 15 frames corresponding to a 3 s observation window are taken, which can cover typical target dynamic segments and facilitate capturing cross-frame associations. Therefore, this paper uses a sliding window to combine consecutive frames into sequence samples for subsequent experiments. Finally, to ensure the comparability of the contrast experiments, Full, Mask-off, Temporal-off, and Aug-off four groups of experiments strictly share the same data division file and consistent division standards, ensuring that the differences only come from the module switches. It should be noted that the baseline uses single-frame slices as the sample unit, and its sample construction and statistical criteria are different from the above sequence samples. Therefore, its support and confusion matrix are only used as single-frame baseline references and are not directly equivalent to the sequence ablation settings for comparison.

### 4.3. Method Comparison

#### 4.3.1. Evaluation Protocol and Data Splitting Strategy

To ensure a rigorous and leakage-free evaluation under highly overlapped sliding-window sampling, we adopt a recording-file-level group-wise splitting strategy together with a fixed hold-out test set. Each raw recording file is treated as an independent group, and all splits are performed at the group level rather than at the windowed-segment level. Consequently, all segments generated from the same recording file are assigned exclusively to a single partition, which prevents near-duplicate segments from the same continuous recording from appearing in both training and evaluation splits and thus mitigates performance overestimation. As shown in Table 2, the raw recording-level dataset statistics are detailed, including the class labels, number of frames, sequence length, and points per frame.

We first construct a strict hold-out test set by selecting a fixed subset of recording groups, with one group per class, as the final test partition. This hold-out test set remains identical across all experiments, including the single-frame baseline, the Full model, ablation variants, and the window and stride sensitivity study. Importantly, the hold-out test set is never used for model selection, hyperparameter tuning, or any training-time decision. It is accessed only for one-time final reporting to provide an unbiased estimate of generalization to unseen recording sessions.

For the Full model, we perform group-wise four-fold cross-validation on the remaining non-test groups. Specifically, the recording groups excluding the fixed hold-out test groups are partitioned into four folds at the group level. For each fold, the model is trained on three folds and validated on the remaining fold, where the validation fold is used for checkpoint selection and optional early stopping when enabled. This file-level cross-validation reduces the risk of over-tuning to a single validation split and provides a more robust estimate of performance stability. After cross-validation, we conduct a final refit. The Full model is retrained using the union of all non-test groups while retaining a small validation subset only for stable checkpoint selection. The refit model is then evaluated on the fixed hold-out test set, and all reported test metrics correspond to this strict hold-out evaluation.

For all comparative settings, including the baseline model, ablation studies, and window and stride sensitivity analysis, we follow the same hold-out test protocol for strict fairness. These comparisons are trained using the final-refit split derived from the above procedure and are evaluated on the unchanged hold-out test set. When varying the window length T and stride s, both specified in the number of radar frames, T denotes the number of frames per sample and s denotes the frame step between consecutive windows. Changing T and s affects the number of generated samples but does not change the underlying group membership of recording files in the training, validation, and test partitions. Therefore, sensitivity results remain directly comparable under an identical hold-out evaluation regime.

A unified training configuration is used across the Full model and all ablation variants, and only the corresponding module is disabled in each ablation setting. To improve robustness, lightweight geometric augmentation is applied to the point cloud coordinates during training, including random in-plane rotation within ±5 degrees and random translation within ±0.1 along the x–y plane. For the Aug-off variant, this augmentation is disabled while all other settings remain unchanged. We optimize the label-smoothed focal loss with ε = 0.02 and γ = 1.5, where class-wise weighting is computed from the training set statistics to alleviate class imbalance. All hyperparameters follow the released training script and the random seed is fixed to 42 for reproducibility.

#### 4.3.2. Baseline

To isolate and quantify the contribution of the proposed spatiotemporal design, we construct a single-frame DGCNN baseline that relies solely on spatial geometry while keeping the data interface consistent with the Full model. Specifically, the baseline disables temporal aggregation by setting the sequence length to one frame, so that no cross-frame information is available and the Transformer-based temporal modeling is removed. This baseline therefore characterizes the discriminative capability achievable from single-frame geometric cues alone, allowing a direct assessment of the gain brought by temporal modeling and the mask-aware design. To ensure a strict and fair comparison, all experiments—including the baseline, ablation variants, and window/stride sensitivity studies—follow the same recording-file-level (group-wise) splitting protocol and share an identical fixed hold-out test set. The hold-out test partition is never used for model selection or hyperparameter tuning and is accessed only for one-time final reporting. In addition, to eliminate implementation-induced bias, the baseline adopts the same padding and mask mechanism as the sequence model: each frame is represented with a fixed-size point set of N = 150 points, and padding points are explicitly marked by setting SNR = −1, while valid points satisfy SNR ≥ 0. The valid-point mask is then used consistently during neighborhood graph construction and feature aggregation, ensuring that performance differences are attributable to modeling choices rather than input formatting. The dataset consists of a child-sized anthropomorphic surrogate, two dogs, and three cats. The surrogate was recorded in sitting and lying postures; therefore, no human subjects were involved in data acquisition. All recordings were collected under the same experimental environment with consistent radar placement and apparatus configuration to minimize scene-induced variations in point cloud statistics. For data construction in the baseline, we directly use single-frame point clouds. A region of interest is first applied according to the radar installation and cage geometry, retaining points within the height range 0 < z < 0.75 m to suppress non-target reflections such as ground and cage-top returns. Each frame is then converted to a fixed-size input with N = 150 points via padding, and the associated valid-point mask prevents padded pseudo-points from affecting kNN graph formation and subsequent feature learning. For the baseline input features, we use only the three-dimensional coordinates $\left(x,\;y,\;z\right)$ as geometric input. The coordinates are centered and scaled to reduce the impact of translation and scale variations across frames. Finally, to measure the intrinsic discriminability of single-frame geometry under a controlled setting, data augmentation is disabled in the baseline experiment. The resulting baseline performance reflects the classification level attainable from spatial-only cues, while the Full model’s improvement quantifies the benefit of incorporating temporal modeling under the same strict hold-out evaluation regime.

**Figure 6:**
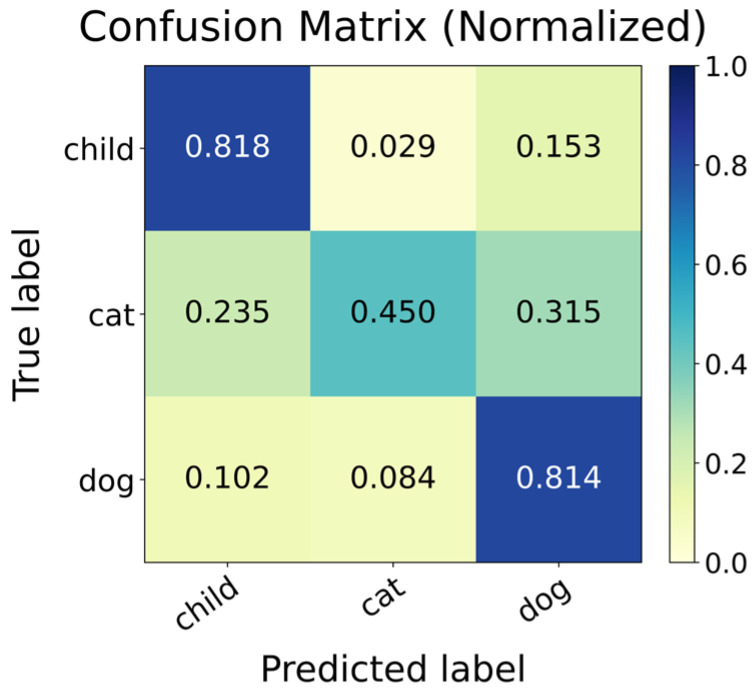
Confusion matrix of baseline (support = 451 samples per class).

Figure 6 presents the normalized confusion matrix of the single-frame DGCNN baseline evaluated on the fixed hold-out test set under the recording-file-level group split. The matrix indicates that most errors concentrate on the Cat class: Cat samples are frequently confused with Child and Dog, whereas Child and Dog show comparatively higher correct recognition. This observation suggests that single-frame geometric cues alone are insufficient to robustly resolve the fine-grained inter-class ambiguity involving Cat, thereby motivating the proposed mask-aware spatiotemporal modeling in the Full framework.

To provide a more comprehensive evaluation, we report additional aggregate metrics on the same fixed hold-out test set, including macro-averaged classification performance and threshold-free ranking metrics. Table 3 summarizes Accuracy, Macro-F1 (Macro-averaged F1-score), and the macro one-vs-rest ROC-AUC (Receiver Operating Characteristic–Area Under the Curve) and PR-AUC (Precision–Recall–Area Under the Curve).

All metrics are computed on the fixed hold-out test set under the recording-file-level group split. Macro-F1 is obtained by averaging the per-class F1-scores with equal weight. We report a 95% confidence interval for Accuracy using the Wilson score interval.

#### 4.3.3. Ablation Experiment

Table 4 summarizes the ablation settings designed to evaluate the contribution of three key components: the valid-point mask, temporal modeling, and data augmentation. The Full setting enables all components, whereas each ablated variant disables exactly one component (Mask-off, Temporal-off, or Aug-off) while keeping the others unchanged. This one-factor-at-a-time design provides a fair and controlled comparison, allowing the performance differences to be directly attributed to the removed module.

**Figure 7:**
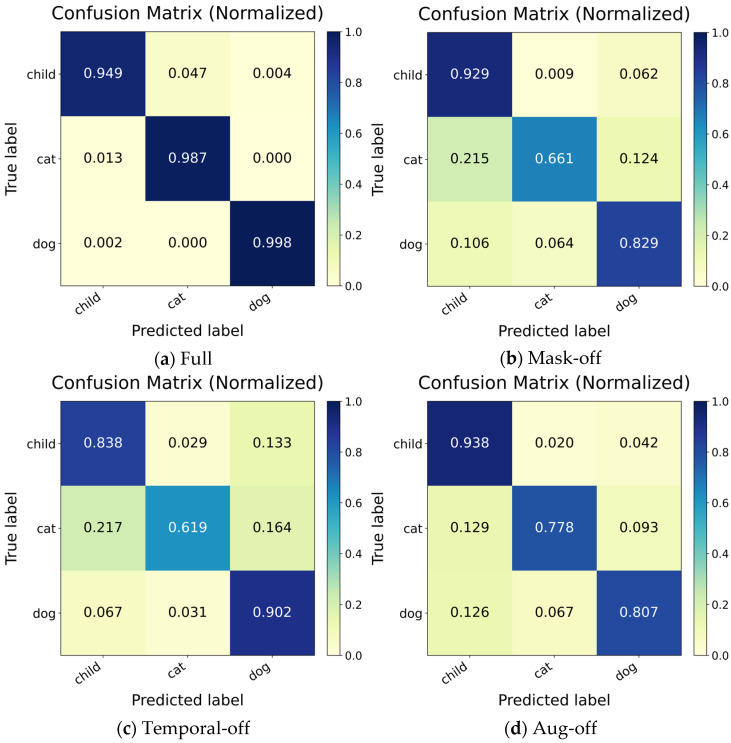
Confusion matrix of ablation experiment (support = 451 samples per class).

Figure 7 presents the normalized confusion matrices for the Full model and three ablation variants in the following order: Full, Mask-off, Temporal-off, and Aug-off. The Full model exhibits strong diagonal dominance, indicating consistently high recall across all three classes with only a small residual confusion of Cat with Child or Dog. This suggests that combining mask-aware spatial aggregation with Transformer-based temporal modeling enables the network to capture both reliable local geometry and discriminative motion patterns over time.

In the Aug-off setting, data augmentation is disabled during training. Compared with the Full model, the main degradation appears in the Cat and Dog classes, implying reduced robustness to minor viewpoint changes, point cloud jitter, and noise fluctuations. This is expected because mild geometric perturbations help the model generalize beyond the specific acquisition conditions, especially for classes with similar spatial signatures.

When temporal modeling is disabled (Temporal-off), misclassifications between Cat and Dog become noticeably more frequent. This indicates that single-frame geometry alone is often insufficient to separate targets with similar body shape and point density, whereas temporal attention across the sequence provides complementary cues by exploiting motion continuity and dynamic patterns. In contrast, removing the valid-point mask (Mask-off) also causes substantial performance deterioration, with errors increasing and spreading across off-diagonal entries. This behavior is consistent with the variable-size and padded representation of mmWave point clouds: once padded pseudo-points are allowed to participate in kNN graph construction and neighborhood feature aggregation, the local topology can be distorted and spurious correlations may be introduced, which degrades the quality of spatial feature learning. Together, the Temporal-off and Mask-off results demonstrate that temporal modeling and mask-aware spatial learning contribute complementary and comparably important benefits: the former reduces ambiguity through dynamics, while the latter stabilizes spatial neighborhood modeling by excluding invalid points.

### 4.4. Sensitivity of Hold-Out Test Accuracy to Sliding-Window Length and Stride

**Figure 8:**
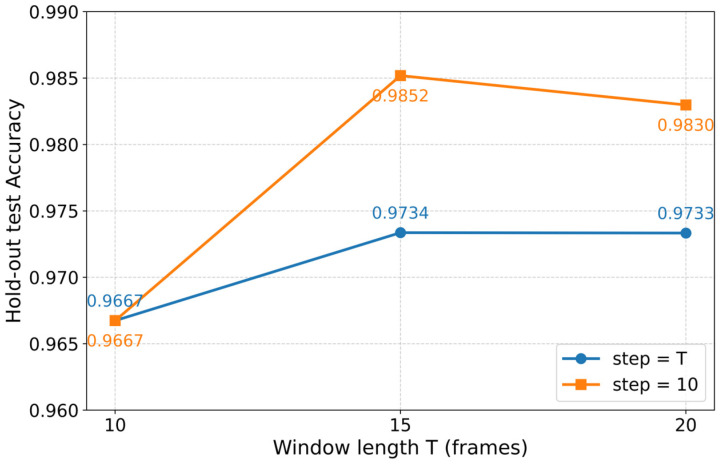
Hold-out test accuracy under different window-length and stride settings.

Figure 8 presents the results of the window sensitivity experiment, showing the impact of different window lengths T and stride step s on model performance. The plot clearly demonstrates that varying T from 10 to 20 frames and adjusting the stride step s has a relatively small effect on the overall recognition accuracy, with a maximum difference less than two percentage points. Specifically, the model performance for T=15, step=10 remains optimal, with an accuracy of 0.9734 for s = T and 0.9852 for s = 10, indicating that the model is stable across different configurations of T and s.

To ensure a fair comparison, the best-performing configuration, T = 15 and s = 10, was selected for subsequent experiments. This configuration balances computational efficiency and performance, as indicated by its consistently high accuracy.

Regarding the data splitting strategy, the fixed hold-out test set was used for final evaluation, with group-wise partitioning applied at the recording-file level to ensure no overlap between the training, validation, and test sets. This method minimizes the risk of information leakage from highly similar segments generated by sliding windows, ensuring robust and reproducible results.

### 4.5. Conclusions

To complement the qualitative evidence from the confusion matrices, we further report quantitative results on the same fixed hold-out test set using macro-averaged metrics and threshold-free indicators, as summarized in Table 5.

Classification performance is noticeably affected by different module settings, as shown in Table 5. The Full model achieves the best overall performance with an Accuracy of 0.978 and a Macro-F1 of 0.978, together with near-saturated ROC-AUC and PR-AUC, indicating stable separability among Child, Cat, and Dog under the strict hold-out evaluation. When the valid-point mask is removed, Accuracy drops to 0.806, corresponding to an absolute decrease of 0.172 compared with the Full model. This degradation indicates that allowing padded or invalid points to participate in neighborhood construction and feature aggregation can distort local topology and introduce spurious correlations, thereby weakening the learned spatial representation. When temporal modeling is disabled, Accuracy further decreases to 0.786, showing that cross-frame dynamics provide important complementary cues for discrimination, particularly for reducing Cat and Dog confusions. When data augmentation is disabled, Accuracy decreases to 0.841, suggesting that lightweight geometric perturbations improve robustness to viewpoint variations, point cloud jitter, and noise fluctuations. Overall, the results demonstrate that valid-point masking and temporal modeling contribute substantial and complementary benefits, while data augmentation provides additional robustness, which is consistent with the error patterns observed in the confusion matrices.

## 5. Discussion

Aiming at millimeter-wave radar point cloud recognition of children and pets in enclosed spaces, this paper proposes a mask-aware spatiotemporal classification framework for point cloud sequences to address sparse sampling, multipath-induced clutter, and fixed-length alignment under large point-count variations. In the spatial branch, local topology learning is used to capture geometric relationships, and a valid-point mask is incorporated to prevent padded points introduced by alignment from participating in neighborhood construction and feature aggregation. In the temporal branch, an attention-based sequence model aggregates cross-frame dynamics to enhance the separability of visually similar classes. Under a strict recording-file-level group-wise split with a fixed hold-out test set, the Full model achieves an Accuracy of 0.978 on the three-class task and demonstrates stable discrimination among Child, Cat, and Dog. Ablation results further confirm the contribution of each component, where Mask-off, Temporal-off, and Aug-off reduce the hold-out Accuracy to 0.806, 0.786, and 0.841, respectively, indicating that valid-point masking and temporal modeling provide substantial and complementary gains, while data augmentation improves robustness to pose perturbations and point cloud jitter.

Despite the strong performance in the current experimental setting, several boundary conditions should be noted for practical deployment. First, the dataset is collected in a single enclosed environment with a limited number of individuals, and the current evaluation focuses on single-target scenarios. In real applications, multiple targets such as a human and a pet may co-exist and partially occlude each other, which can cause merged or fragmented point clusters and lead to degraded classification reliability. Second, variations in installation height, sensor tilt, and surrounding reflective surfaces can alter the multipath pattern and the point distribution, potentially increasing background clutter and changing the effective field of view. Third, different ground materials and reflection conditions may shift the clutter level and the point-count statistics, which can affect fixed-length alignment and, consequently, neighborhood topology estimation. Future work will extend the dataset to multiple environments and installation configurations, and will include qualitative failure cases accompanied by quantitative performance ranges under these boundary conditions. In addition, we will explore integrated multi-target detection, tracking, and association to enable simultaneous human–pet recognition, and investigate lightweight architectures and deployment-oriented inference optimization to improve real-time performance for practical on-device use.

## Figures and Tables

**Table 1:** Radar parameters.

Parameters	Symbol	Value
MIMO array	*Tx/Rx*	3Tx/4Rx
Frame rate	*f_frame_*	5 fps
Number of cycles per frame	*NumLoops*	32
Chirp per frame	*N_chirp_*	32 × 3=96
Number of range bins	*N_r_*	64
Sweep bandwidth	*B*	2 GHz
Range resolution	$\Delta R=\frac{c}{2B}$	0.075 m
Sampling frequency	*f_s_*	25 MHz
Azimuth/elevation	*(θ, ϕ)*	[−60°, 60°]
Constant false-alarm rate	*P_fa_*	0.005

**Table 2:** Raw recording-level dataset statistics.

Parameters	Value
Class labels	Child/Cat/Dog
Number of frames	19,216/40,625/19,573
Sequence length	15 frames
Points per frame	150/frame

**Table 3:** Class-wise performance metrics.

Accuracy	95% CI	Macro-F1	ROC-AUC	PR-AUC
0.694	[0.669, 0.718]	0.682	0.852	0.748

**Table 4:** Ablation experimental protocol.

	Valid-Point Mask	Temporal Model	Data Augmentation
Full	✓	✓	✓
Mask-off	✗	✓	✓
Temporal-off	✓	✗	✓
Aug-off	✓	✓	✗

**Table 5:** Quantitative evaluation metrics on the fixed hold-out test set.

Settings	Accuracy	95% CI	Macro-F1	ROC-AUC	PR-AUC
Full	0.978	[0.968, 0.984]	0.978	0.998	0.997
Mask-off	0.806	[0.784, 0.827]	0.803	0.942	0.894
Temporal-off	0.786	[0.764, 0.807]	0.782	0.931	0.885
Aug-off	0.841	[0.821, 0.860]	0.840	0.951	0.918

## Data Availability

The data presented in this study are available on request from the corresponding author. The data are not publicly available due to privacy and project restrictions.

## Abbreviations

The following abbreviations are used in this manuscript:

mmWave	Millimeter-wave
FMCW	Frequency-Modulated Continuous Wave
IF	Intermediate Frequency
ADC	Analog-to-Digital Converter
FFT	Fast Fourier Transform
RD Map	Range–Doppler Map
CFAR	Constant False Alarm Rate
CA-CFAR	Cell-Averaging CFAR
CUT	Cell Under Test
AoA	Angle of Arrival
TDM-MIMO	Time-Division Multiplexing Multiple-Input Multiple-Output
Capon/MVDR	Capon (Minimum Variance Distortionless Response) beamforming
SNR	Signal-to-Noise Ratio
ROI	Region of Interest
kNN	K-Nearest Neighbors
DGCNN	Dynamic Graph Convolutional Neural Network
EdgeConv	Edge Convolution
MLP	Multilayer Perceptron
MSA	Multi-Head Self-Attention
FFN	Feed-Forward Network
LN	Layer Normalization
ROC-AUC	Area Under the Receiver Operating Characteristic Curve
PR-AUC	Area Under the Precision–Recall Curve
